# Supplementary figures and images for: Optogenetic mapping of feeding and self-stimulation within the lateral hypothalamus of the rat
Source: PLoS One. 2020 Jan 27;15(1):e0224301. doi: 10.1371/journal.pone.0224301 (PMC6984703; doi:10.1371/journal.pone.0224301)

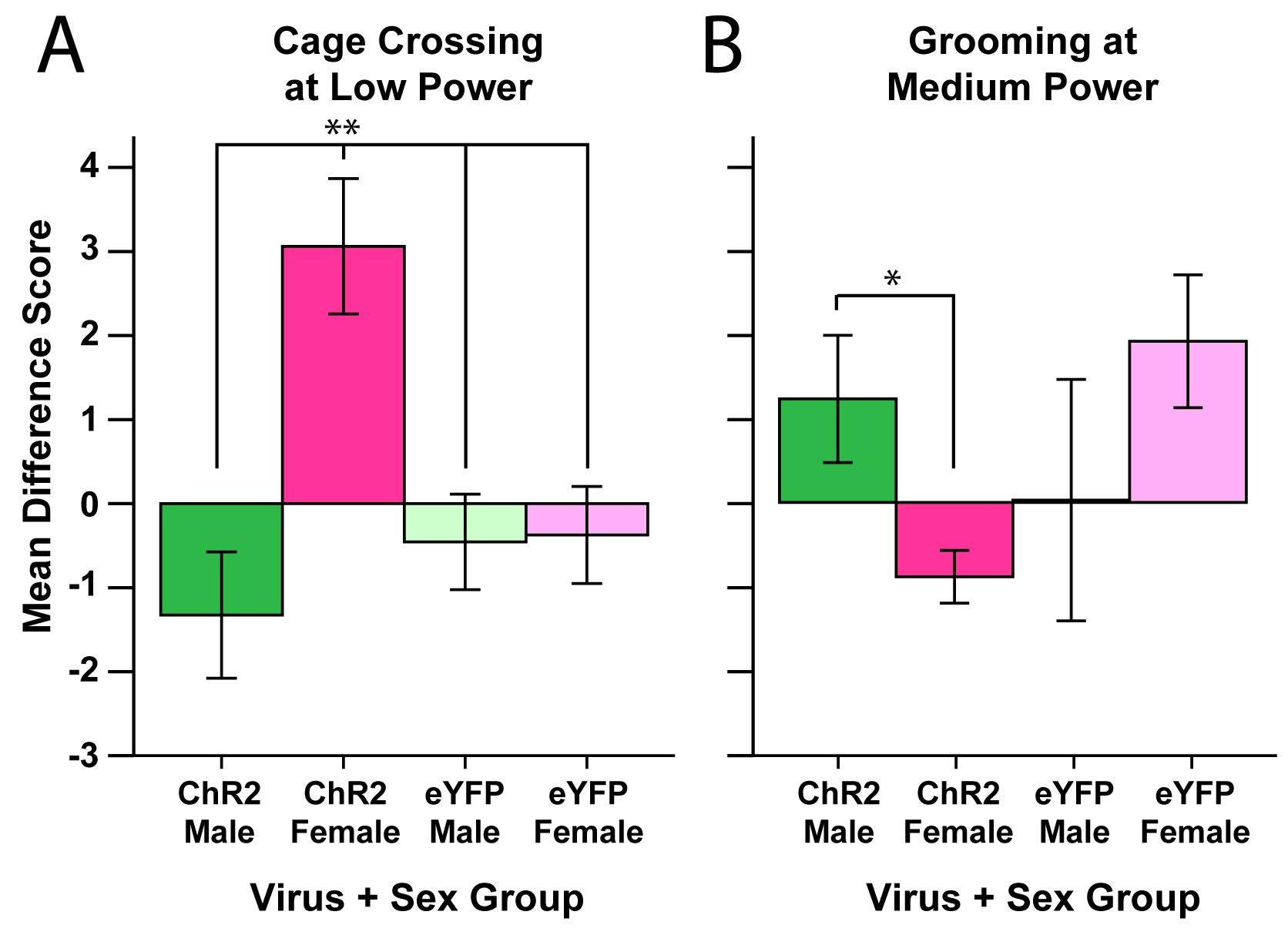

Supplement: S1 Fig — Behavioral difference scores (laser period score minus interim period score) for male and female ChR2 and eYFP rats engaging in A. cage crossing during low laser power output or B. grooming during medium laser power output. (TIF) [file pone.0224301.s001.tif]
